# Correction to ‘Hidden Mould in the Air: A Practical Narrative Mini‐Review of Invasive Mould Infection Prevention for High‐Risk Patients and Clinicians’

**DOI:** 10.1111/myc.70231

**Published:** 2026-10-22

Bauer A , Lass‐Flörl C , Steixner S , Cornely OA , Reinhold I , Walti L , et al. “Hidden Mould in the Air: A Practical Narrative Mini‐Review of Invasive Mould Infection Prevention for High‐Risk Patients and Clinicians,” Mycoses 69, no. 9 (2026): e70224, 10.1111/myc.70224.PMC1355920542717664

The name of the author, Oliver A. Cornely, was incorrect and has been updated from “Cornely O” to “Cornely OA”.

The online version of this article has been corrected accordingly.

We apologize for this error.

